# Inhibiting glutaminase in acute myeloid leukemia: metabolic dependency of selected AML subtypes

**DOI:** 10.18632/oncotarget.12944

**Published:** 2016-10-27

**Authors:** Polina Matre, Juliana Velez, Rodrigo Jacamo, Yuan Qi, Xiaoping Su, Tianyu Cai, Steven M. Chan, Alessia Lodi, Shannon R. Sweeney, Helen Ma, Richard Eric Davis, Natalia Baran, Torsten Haferlach, Xiaohua Su, Elsa Renee Flores, Doriann Gonzalez, Sergej Konoplev, Ismael Samudio, Courtney DiNardo, Ravi Majeti, Aaron D. Schimmer, Weiqun Li, Taotao Wang, Stefano Tiziani, Marina Konopleva

**Affiliations:** ^1^ Departments of Leukemia, The University of Texas MD Anderson Cancer Center, Houston, TX, USA; ^2^ Bioinformatics and Computational Biology, The University of Texas MD Anderson Cancer Center, Houston, TX, USA; ^3^ Department of Medicine, Division of Hematology, Cancer Institute, and Institute for Stem Cell Biology and Regenerative Medicine, Stanford University School of Medicine, Stanford, CA, USA; ^4^ Department of Nutritional Sciences, The University of Texas at Austin, Austin, TX, USA; ^5^ Lymphoma, Division of Cancer Medicine, The University of Texas MD Anderson Cancer Center, Houston, TX, USA; ^6^ CEO of MLL Munich Leukemia Laboratory, Munich, Germany; ^7^ Molecular & Cellular Oncology, The University of Texas MD Anderson Cancer Center, Houston, TX, USA; ^8^ Hematopathology, The University of Texas MD Anderson Cancer Center, Houston, TX, USA; ^9^ The Centre for Drug Research and Development Biologics, Vancouver, British Columbia, Canada; ^10^ Medical Biophysics, Princess Margaret Hospital / Ontario Cancer Institute, University Health Network, Toronto, Ontario, Canada; ^11^ Analytical Chemistry, Pharmacology, Spectroscopy, Calithera Biosciences, South San Francisco, CA, USA

**Keywords:** leukemia, metabolism, glutamine, microenvironment, differentiation therapy

## Abstract

Metabolic reprogramming has been described as a hallmark of transformed cancer cells. In this study, we examined the role of the glutamine (Gln) utilization pathway in acute myeloid leukemia (AML) cell lines and primary AML samples. Our results indicate that a subset of AML cell lines is sensitive to Gln deprivation. Glutaminase (GLS) is a mitochondrial enzyme that catalyzes the conversion of Gln to glutamate. One of the two GLS isoenzymes, GLS1 is highly expressed in cancer and encodes two different isoforms: kidney (KGA) and glutaminase C (GAC). We analyzed mRNA expression of *GLS1* splicing variants, *GAC* and *KGA,* in several large AML datasets and identified increased levels of expression in AML patients with complex cytogenetics and within specific molecular subsets. Inhibition of glutaminase by allosteric GLS inhibitor *bis*-2-(5-phenylacetamido-1, 2, 4-thiadiazol-2-yl) ethyl sulfide or by novel, potent, orally bioavailable GLS inhibitor CB-839 reduced intracellular glutamate levels and inhibited growth of AML cells. In cell lines and patient samples harboring IDH1/IDH2 (Isocitrate dehydrogenase 1 and 2) mutations, CB-839 reduced production of oncometabolite 2-hydroxyglutarate, inducing differentiation. These findings indicate potential utility of glutaminase inhibitors in AML therapy, which can inhibit cell growth, induce apoptosis and/or differentiation in specific leukemia subtypes.

## INTRODUCTION

**Figure 1:**
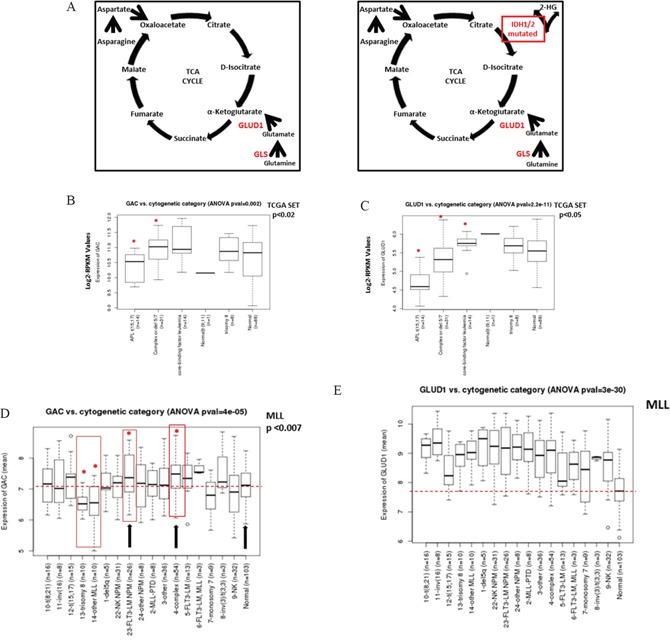
Expression of *GAC* and *GLUD1* in TCGA and MLL AML datasets respectively **A.** Principal genes related to Gln metabolism. **B.** Boxplot comparing expression values of *GAC* and **C.** *GLUD1* from the TCGA AML dataset. Expression represents batch-effect adjusted, normalized, log2-transformed RPKM (Reads per Kilobase of transcript per Million mapped reads) values. **D.** *GAC* and **E.** *GLUD1* expression from the MLL AML dataset in different cytogenetic abnormality categories. The p-values were determined by ANOVA. Asterisk and red boxes denote categories with statistically significant higher *GAC* expression compared to healthy normal donors; median *GAC* in normal controls is shown by dotted red line. NK NPM, AML with normal karyotype, NPM-mutant; FLT3-LM NPM, AML with normal karyotype, FLT3-ITD length mutation and NPM-co-mutated; NK, normal karyotype AML.

Metabolic deregulation has been denoted as a hallmark of cancer. Glutamine is a non-essential amino acid that plays a unique role in the metabolism of proliferating cancer cells, providing building blocks to sustain cell proliferation and regulating redox homeostasis and signal transduction pathways. Glutaminase (GLS) and glutamate dehydrogenase 1 (GLUD1) are mitochondrial enzymes that catalyze the first and second steps of Gln catabolism. GLS catalyzes the conversion of Gln to glutamate (Glu), while GLUD1 catalyzes the conversion of Glu to alpha-ketoglutarate (α-KG) and ammonia (Figure 1A). GLS gene encodes for two tissue-specific isoenzymes “kidney-type” (GLS, alias GLS1: glutaminase, Homo sapiens, Gene ID: 2744) and “liver type” (GLS2: glutaminase 2, Homo sapiens, Gene ID: 27165); each one existing in two different isoforms: kidney (KGA) and glutaminase C (GAC) for GLS1, and liver (LGA) and Glutaminase B (GAB) for GLS2. The GAC isoform has been found highly expressed in different types of cancer including lymphoma, glioma, breast, pancreatic etc. Inhibition of glutaminase has been recently reported by several groups as an attractive therapeutic approach in various cancers [1–3], and studies using small molecule inhibitors to block its enzymatic activity or genetic knockdown have demonstrated antitumor activity in models of lymphoma and glioma, and of breast, pancreatic, and renal cancer [2–3].

*Bis*-2-(5-phenylacetamido-1,2,4-thiadiazol-2-yl)ethyl sulfide (BPTES) is a reversible allosteric glutaminase inhibitor [4–6]. CB-839 is a novel, potent, orally bioavailable GLS inhibitor that has been reported to have antiproliferative activity in a panel of triple negative breast cancer cell lines [3] and most recently in acute myeloid leukemia (AML) [7]. BPTES and CB-839 share a mechanism of action through allosteric inhibition of glutaminase, selectively inhibiting both GLS isoforms but not GLS2. However, CB-839 is characterized by greater nanomolar potency and better oral bioavailability than BPTES [3].

While the metabolic alterations in AML are poorly characterized, recent findings indicate that leukemic cells are able to utilize Gln as a carbon source for energy generation and growth [8, 9]. A subset of leukemia cells with mutations in the metabolic enzymes isocitrate dehydrogenase 1 (IDH1) and 2 (IDH2), and IDH1-mutant glioma cells have been shown to be particularly sensitive to glutaminase inhibition [10]. The frequency of mutations in IDH1 and IDH2 in *de novo* AML is 20%. Wild-type IDH1/2 catalyzes the conversion of isocitrate to α-KG in cytosolic and mitochondrial compartments; in contrast, mutant IDH1 and IDH2 result in a neoenzymatic activity, reducing α-KG and producing 2-hydroxyglutarate (2-HG). Gln has been shown to be a cellular source of α-KG, converted further to 2-HG by mutant IDHs [11]. Emadi *et al*. showed that inhibition of glutaminase by BPTES is deleterious to cellular metabolism and growth of mutant IDH cells [12]. However, no data are available about *GLS* expression or functional significance in other AML subtypes.

In this study, we characterized the anti-AML efficacy of the novel GLS inhibitor CB-839 with the goal of elucidating the role of glutamine in leukemia. Our findings indicate that a subset of AML cell lines and primary AML cells are sensitive to Gln deprivation and to inhibition of glutaminase by BPTES or CB-839. In AML harboring IDH1/2 mutations, CB-839 reduced the levels of oncometabolite 2-HG and induced myeloid differentiation.

## RESULTS

### GLS and GLUD1 transcripts are overexpressed in selected cytogenetic and molecular genetic subgroups of AML

First, we analyzed expression of genes related to Gln metabolism (Figure 1A) in The Cancer Genome Atlas (TCGA) AML cohort. In this AML dataset, the mean expression value of *GLS* was within the upper 4% of all genes, and expression of *GLUD1* was within the upper 6% of all genes (Supplementary Table S1). The expression level of *GLS* was significantly higher than that of *GLS2*, with mean expression values (log2-RPKM) of 4.69 and 0.54 (data not shown), respectively. Among *GLS* isoforms, the expression level of *GAC* was much higher than that of *KGA*, 10.72 vs 0.11, *t*-test p<2.2×10^−16^ (Supplementary Figure 1A).

We next analyzed expression of *GLS* within different cytogenetic and molecular cohorts. While expression of *GLS2* did not differ significantly between cytogenetic abnormality categories (Supplementary Figure 1B), pairwise comparison of *GLS* expression in different cytogenetic abnormality categories using the two-sample Wilcoxon test showed significant difference between categories (Supplementary Figure 1C). In particular, expression of the *GAC* transcript was significantly higher in AML with complex or del 5/7 cytogenetics (n=31) and in core-binding factor AML (representing t(8;21) and inv(16)) (CBF-AML, n=14) than in normal karyotype AML (n=88, p=0.0187 and 0.00184, respectively; Figure 1B). Pairwise comparison of *KGA* expression likewise showed higher expression in the complex or del 5/7 cytogenetic subgroup than in diploid AML (p=0.01, not shown). Consistent with the reported role of Myc in transcriptional regulation of mitochondrial glutaminolysis [1], *GAC* mRNA levels positively correlated with *c-MYC* mRNA levels (Pearson correlation coefficient 0.47, p=1×10^−10^; Supplementary Figure 1D).

Expression of *GLUD1* was significantly different between cytogenetic abnormality categories (ANOVA p=2.2×10^−11^; Figure 1C). Pairwise comparison of *GLUD1* expression in different cytogenetic abnormality categories using the two-sample Wilcoxon test showed significantly higher expression of *GLUD1* in CBF-AML than in normal karyotype cases (p=0.045). In contrast, the expression of *GLUD1* in acute promyelocytic leukemia (t(15; 17)) and cases with complex or del 5/7 cytogenetics was lower than in normal karyotype AML (p= 1.3×10^−7^ and 0.023, respectively).

We next compared the expression of *GLS*/*GLS2* and of *GLUD1* by mutation status of genes *IDH1*/*2*, *NPM1*, *FLT3*, and *KRAS*/*NRAS*. The expression of *GLS* mRNA was significantly higher in *NPM1* wild-type (WT-*NPM1*) AML than in mutant *NPM1* (mut-*NPM1*) AML (*t*-test p=0.03; Supplementary Figure 1E); all other comparisons were not significant. Among splicing isoforms, *GAC* was not differentially expressed, while *KGA* expression was higher in WT-*NPM1* AML than in mut-*NPM1* AML (p=0.027; Supplementary Figure 1F). The expression of *GLUD1* was significantly higher in mut-*FLT3* than in WT-*FLT3* AML (*t*-test p=0.028) and in mut-*NPM1* AML than in WT-*NPM1* AML (*t*-test p=0.0038; not shown).

Since the TCGA dataset does not contain any normal BM samples, we next compared the expression of *GAC*, *KGA*, and *GLUD1* in the Munich Leukemia Laboratory (MLL) AML dataset [13]. Gene expression was determined by using oligonucleotide microarrays (HG-U133 Plus 2.0, Affymetrix) in 288 AML and 103 normal karyotype samples (donors with healthy BM and non-leukemia conditions) [13]. Consistent with TCGA data, *KGA* expression was lower than *GAC* expression (data not shown). In comparison to normal controls, the *GAC* transcript was significantly overexpressed in AML with complex cytogenetics (p=0.0007) and moderately higher in AML with *FLT3-ITD/NPM1* gene mutations (p=0.0077); it was significantly lower in AML with trisomy 8 (p=0.001; Figure 1D). When the analysis was repeated using normal karyotype AML as a comparison, *GAC* expression was likewise significantly higher in AML with complex cytogenetics or *FLT3-ITD/*mut*NPM1* (p=0.006 and 0.001, respectively, not shown). Strikingly, the expression of *GLUD1* was much higher in the majority of AML categories than in normal karyotype BM (ANOVA p=3×10^−30^; Figure 1E). Consistent with TCGA dataset results, *GLUD1* expression was higher in CBF-AML than in diploid cases (p = 0.02) and in mut-*NPM1* (p=0.01) but not FLT3-ITD AML.

In a gene expression dataset of various AML subtypes (n = 529) described previously by Verhaak *et al*. [14], higher expression of *GAC* was confirmed in AML with t(8;21) (n=37, 1.27-fold, p=0.00077), *FLT3-ITD* AML (n=143, 1.25-fold, p=1.84×10^−7^) and interestingly AML with silenced *CEBPA* (unmutated, with decreased *CEBPA* mRNA levels [15]) (n=10, 1.7-fold, p=2.92×10^−7^) than in the remaining AML. *GLUD1* was highly expressed in AML with t(8;21) (1.15-fold, p=0.004). These findings indicate high expression of *GLS* in selected cytogenetic and molecular subtypes of AML.

### Targeting Gln utilization with a GLS inhibitor inhibits AML cell growth

**Figure 2:**
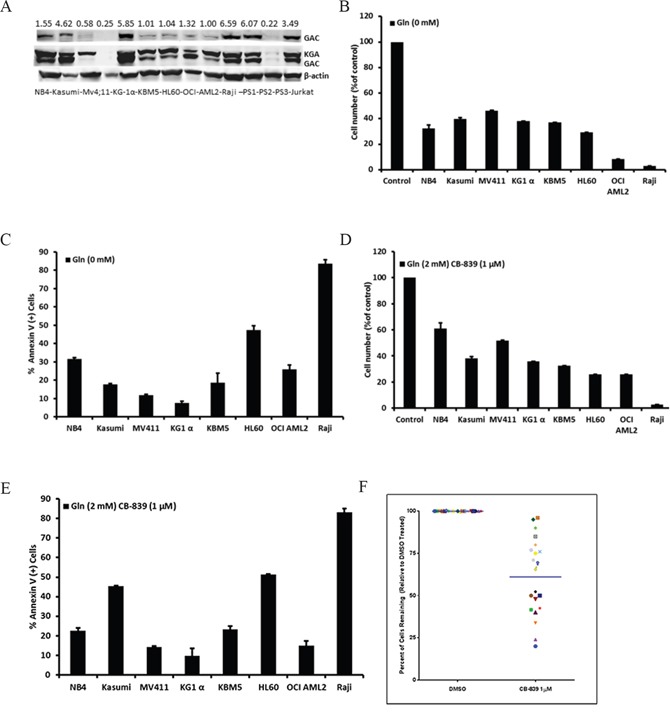
Viability and apoptosis of leukemic cell lines and AML primary samples after Gln deprivation and GLS1 inhibition by CB-839 **A.** Expression of GLS isoforms, GAC and KGA in leukemic cell lines. AML cell lines showed high expression levels of the KGA and GAC isoform. The expression of the full length GLS protein KGA (669 aa) and the shorter isoform GAC (598 aa) was determined in a panel of leukemia (n=10) by Western blotting using GAC- specific antibody (top panel) and an antibody that binds to both GAC and KGA isoforms (bottom panel). **B-D.** The percentage of viable leukemia cells among cells treated with Gln deprivation or GLS1 inhibition by CB-839 was normalized to the percentage of viable cells in control samples measured after 3 days of culture by multicolor flow cytometry. **C-E.** Levels of specific apoptosis in CB-839–treated cell lines relative to control, measured by annexing V flow cytometry. **F.** The percent of viable AML blast cells (blast gate determined based on CD45/side scatter) in treated samples normalized to viable AML blasts in controls was measured after 72 hours by flow cytometry (n=21).

We analyzed expression of the full-length GLS protein KGA (669 aa) and the shorter isoform GAC (598 aa) in a panel of leukemia cell lines (n = 10) and in 3 primary AML samples using GAC (Proteintech 19958-1-AP 1:1000) and KGA-GAC (Abcm 156876) specific antibodies (Figure 2A). Molecular characterization of AML cell lines and primary sample information is listed in Supplementary tables 2 and 3, respectively. Consistent with gene expression data, AML cell lines and 2 of the 3 primary AML samples tested exhibited high levels of both KGA and GAC isoforms (Figure 2A, top panel). To functionally characterize the importance of Gln for proliferation and survival of AML cells, we next examined the effects of Gln withdrawal or pharmacological inhibition of glutaminase on cellular growth in a panel of acute leukemia cell lines. In all cell lines tested, Gln deprivation caused steep (>60%) decreases in viable cell numbers (Figure 2B) and induced apoptosis to different extents (Figure 2C). In turn, inhibition of GLS with CB-839 (1 μM) predominantly inhibited cellular growth (~40%) (Figure 2D), while induction of apoptosis was seen in some cell lines, such as NB4, Kasumi, KBM5, HL-60, and Myc-driven Burkitt lymphoma cell line Raji, used as a positive control (Figure 2E) Notably, GAC expression was elevated in Kasumi cells harboring t(8;21) and HL-60 AML cells with complex cytogenetics, exhibited higher apoptosis induction in response to CB-839. Similar results were observed when GLS was inhibited with BPTES (Supplementary Figure 2A, 2B). Notably, greater sensitivity to CB-839 correlated with greater sensitivity to Gln deprivation (r=0.774, p=0.024 for viability and r=0.968, p<0.01 for apoptosis; Supplementary Figure 2C-2D).

Next we tested the efficacy of CB-839 in primary samples from patients with relapsed/refractory AML (n=21). Consistent with the results in AML cell lines, CB-839 (1 μM) significantly decreased viable cell number in the majority of samples tested without significant apoptosis induction (Figure 2F and Supplementary Figure 2E). Overall, these data are consistent with recently reported findings in AML cells [7].

### CB-839 blocks Gln utilization in AML cells

**Figure 3:**
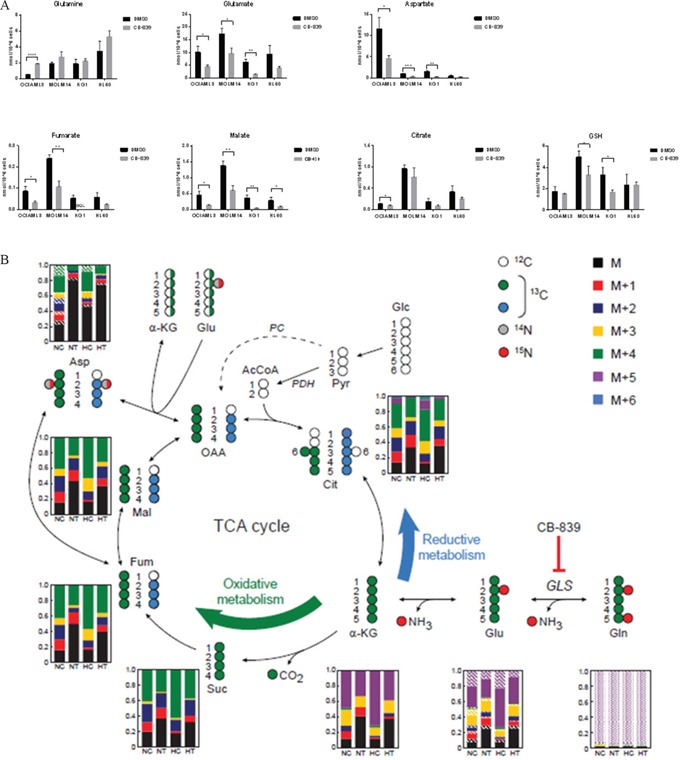
CB-839 blocks Gln utilization in AML cells **A.** CB-839 treatment induced moderate accumulation of Gln and decreased cellular levels of glutamate Glu, aspartate, TCA acid cycle intermediates, and GSH in AML cell lines. **B.** In SIRM analysis, incorporation of ^13^C_5_, ^15^N_2_ Gln into Krebs cycle intermediates *via* oxidative and reductive metabolism of Gln. Molecular structures of Krebs cycle intermediates are represented as follows: white circles represent ^12^C, green and blue circles indicate ^13^C atoms, and grey and red circles correspond to ^14^N and ^15^N atoms, respectively. For simplicity, only the first round of the Krebs cycle is shown and the contribution of carbons derived from glucose through pyruvate carboxylase (PC; dashed arrow) is not shown. The bar graphs represent the relative fractions of ^13^C- and/or ^15^N-enriched compounds measured by LC-MS. Average levels from four replicate samples are shown. Solid color bars indicate different units of ^13^C enrichment (as per legend) without ^15^N enrichment, oblique lines and dotted areas correspond to ^15^N enrichment of one and two N atoms, respectively. NC and HC, control cells under normoxic and hypoxic conditions; NT and HT, cells treated with CB-839 under normoxic and hypoxic conditions, respectively.

We next examined the effect of glutaminase inhibition on the levels of intracellular metabolites in various AML cell lines (OCI-AML3, MOLM-14, HL-60 and KG-1), by using liquid chromatography–mass spectroscopy (LC-MS; Figure 3A). CB-839 treatment caused depletion of Glu (GLS metabolic product) and moderate accumulation of Gln (substrate), statistically significant in OCI-AML3. CB-839 further reduced concentrations of a number of key metabolites downstream of Glu, including intracellular aspartate (produced from Glu via aspartate aminotransferase activity); tricarboxylic acid (TCA) cycle intermediates fumarate, malate and citrate (produced from Glu-derived α-KG); and glutathione (GSH) synthesized from Glu. Similar metabolic effects were observed in OCI-AML3 cells treated with BPTES, which caused decreases in Glu, aspartate, and Krebs cycle intermediates (Supplementary Figure 3A).

Stable isotope-resolved metabolomics (SIRM) analysis with ^13^C_5_, ^15^N_2_-Gln in HL-60 cells indicated that treatment with CB-839 severely hindered Gln anaplerosis to similar extents under normoxic or hypoxic conditions (Figure 3B). Moreover, Gln is predominantly used to carry out oxidative metabolism. While the enrichment fraction in each of the Krebs cycle intermediate compound pools dropped from about 75–80% to 50–60% in the treated samples under both normoxic and hypoxic conditions, the enriched fraction of aspartate in treated cells dropped dramatically (to approximately 20% or less of the pool), suggesting that leukemia cells strictly depend on Krebs cycle-derived oxaloacetate transamination for the generation of aspartate.

Reduction in intracellular concentrations of Krebs cycle intermediates suggests that glutaminase inhibition reduces anaplerotic carbon flow through the Krebs cycle and may impair the molecular reduction of oxygen. We measured the oxygen consumption rates in OCI-AML3 cells using a Seahorse Bioanalyzer. Glutaminase inhibition with CB-839 (1 μM), or BPTES (20 μM) Supplementary Figure 3B caused a decrease of the basal and maximal respiratory capacity, supporting the notion that glutamate anaplerosis supports the generation of reducing equivalents (NADH, FADH_2_) by the Krebs cycle.

### CB-839 modulates intracellular metabolites and reduces 2-HG concentration in IDH1/IDH2 mutant leukemic cells

**Figure 4:**
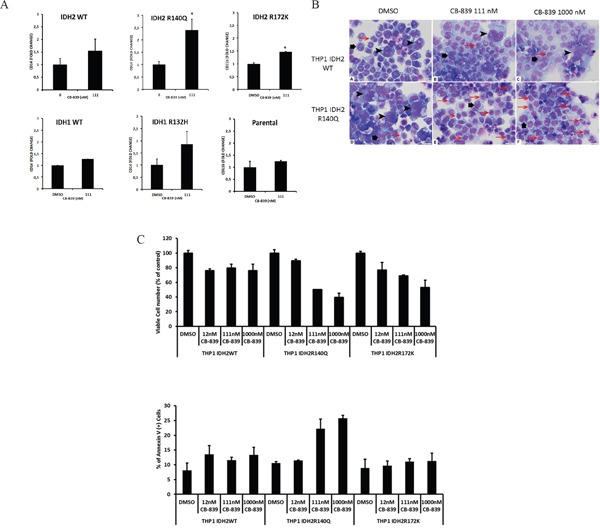
CB-839 treatment promotes growth arrest and differentiation in THP-1 cell lines with mutant IDH1/2 **A.** wild-type IDH1/2 or doxycycline-inducible mutants IDH2-R140-R172 were exposed to CB-839 for 8 days. Expression of differentiation marker CD14 was determined through multicolor flow cytometry. **B.** Morphological signs of differentiation induced by CB-839 treatment in THP-1 cells stably transduced with WT IDH2 or IDH2-R140Q were determined with hematoxylin-eosin staining after 8 days of treatment. The composite figure illustrates representative cytospin preparations of IDH2 WT (A-C) and mutant IDH2-R140 (D-F) cells. The control samples (A, D) demonstrated a mixture of predominantly giant anaplastic cells (arrowheads) and moderately differentiated medium-sized cells (large arrows) with relatively few small differentiated cells (small red arrows). Treatment with CB-839 111 nM (B) or CB-839 1000 nM (C) did not change IDH2 WT cell composition of the sample. In contrast, treatment with CB-839 111 nM (E) or CB-839 1000 nM (F) resulted in significant differentiation of IDH2-R140–mutated cells, which is supported by the disappearance of giant anaplastic cells and marked increase in small differentiated cells. **C.** Viability and apoptosis in THP-1 cells transduced with WT-IDH2, IDH2-R140Q, or IDH2-R172K cells were measured by flow cytometry.

Mutations in the metabolic enzymes IDH1 and IDH2 are frequently found in glioma, cholangiocarcinoma, T-cell lymphomas, thyroid cancer, chondrosarcoma, and AML. Gln, through Glu, is a precursor for cellular α-KG, which can undergo further metabolism through the Krebs cycle or be further metabolized to 2-HG by mutant IDHs. In THP-1 cell lines stably transduced with doxycycline-inducible mutant IDH1-R132H or IDH2-R140Q constructs [16], CB-839 exposure for 4 days reduced intracellular 2-HG oncometabolite levels by >50% (Supplementary Figure S4). It is likely that substrates other than glutamine are capable to contribute towards 2-HG synthesis in IDH-mutant in AML cells, explaining only partial inhibition of 2-HG production with CB-839. Nevertheless, this reduction was associated with induction of differentiation marker CD14 and morphological signs of differentiation in CB-839–treated IDH2-R140Q, IDH2-R172, and IDH1-R132 cells but not in WT-IDH2, WT-IDH1, or parental THP-1 control cells (Figure 4A-4B). In turn, cells expressing mut-IDH2-R140Q or IDH2-R172 were significantly more sensitive to CB-839 (1 μM) than WT-IDH2 cells (Figure 4C).

**Figure 5:**
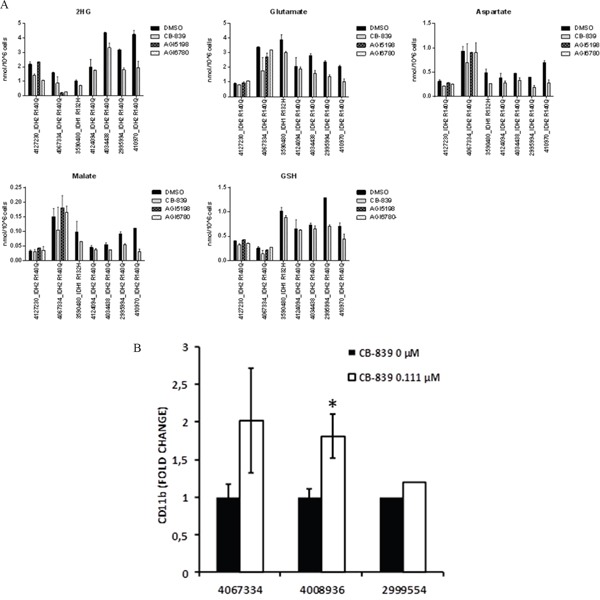
CB-839 induces metabolic changes in IDH1/2 mutant AML patient samples **A.** CB-839 treatment decreased cellular levels of 2-HG, Glu, aspartate, malate, and GSH in IDH1/IDH2–mutant AML patient samples. Intracellular metabolite levels were measured in primary AML samples after treatment with CB-839 (1 μM), IDH1 inhibitor AGI-5198 (500 nM), or IDH2 inhibitor AGI-6780 (500 nM) for 24 hours (4127230; 3590480; 4124094) or 96 hours (4067334, 4034438; 2995994). **B.** Flow cytometry analysis in IDH2-R140Q (4067334)–, IDH1-R132C (4008936), and IDH2-R140Q (2995994) mutated AML samples showed an increase in a fraction of CD11b+ cells after treatment with CB-839 (0.111 μM). Because of limited sample size, triplicate measurements could not be performed in sample number 2995994.

In mut-IDH1 or mut-IDH2 primary AML samples, CB-839 caused moderate reductions (range 12-58%, average 35.6±15.1%, p=0.015) in 2-HG levels (Figure 5A), in concert with expected decreases in concentrations of Glu, aspartate, malate, and GSH. In two mut-IDH2 samples, IDH2 inhibitor AGI6780, as expected, reduced 2-HG levels without affecting Gln-related metabolites. CB-839 induced myeloid differentiation in three of four mut-IDH samples tested: 4067334 (IDH2-R140Q mutation), 4008936 (IDH1-R132C mutation), and 2999554 (IDH2-R140Q mutation). In sample 4067334 harboring the IDH2-R140Q mutation, CB-839 exposure caused reduction of 2-HG by 45% and resulted in a higher proportion of cells expressing myeloid differentiation marker CD11b (2-fold; Figure 5B).

## DISCUSSION

In this study, we report a comprehensive analysis of expression of the *GLS* and *GLUD1* genes in three separate AML datasets. Although different technologies (Affymetrix arrays and RNAseq data in TCGA) were used [13, 14] and annotation of genetic categories differed somewhat, several conclusions can be derived from this analysis. First, *GLS* isoform *GAC* and *GLUD1* were highly expressed in AML, while isoform *KGA* was expressed at a low level, findings consistent with data on other malignancies. *GAC* was overexpressed in AML with complex cytogenetics when compared with cases with normal BM [13] in the MLL dataset or normal karyotype AML in the TCGA dataset. Cases with a *FLT3* mutation tend to have higher expression of *GAC*, although this was not replicated in the TCGA dataset. Likewise, *GAC* expression was higher in cases with t(8;21) in the Verhaak *et al*. [14] dataset and the TCGA dataset. Expression of *GLUD1* was significantly higher in the majority of AML subtypes compared to normal healthy BM, and it was higher in CBF-AML in the TCGA and Verhaak *et al*. [14] datasets than other subtypes. These findings suggest that specific AML subtypes express high levels of genes required for Gln utilization as an energy source in Krebs cycle entry.

The targeting of metabolic processes has emerged as a novel, promising approach in cancer treatment, mostly because of the increased metabolic influxes, including in glucose and Gln, that are required for optimal proliferation and survival of cancer cells [9]. Until recently, most therapies under development focused on glucose metabolism, whereas therapies targeting Gln are now entering the therapeutic arena. To probe for the functional role of Gln in AML, we utilized the novel selective nanomolar potency GLS inhibitor CB-839, which is currently undergoing clinical evaluation as both a single agent and in rational combinations in solid tumors and hematologic malignancies. In agreement with recently published data, our AML cell line data indicate that AML cells utilize Gln as an energy source for proliferation, and this reliance translates into sensitivity to CB-839. We demonstrated that the absence of Gln in the medium or decrease of its catabolism through glutaminase inhibition induced cell growth suppression (>40%) in all cell lines tested and induced apoptosis (>30%) in two AML cell lines (Kasumi-1, HL-60) and one ALL cell line (Raji).

Metabolic analysis demonstrated expected decreases in Glu and aspartate, reductions of Krebs cycle metabolites, and modest decreases in GSH upon treatment with glutaminase inhibitors (BPTES or CB-839). These data are reinforced by similar findings in different cancer models for glutaminase knockdown (by siRNA) and BPTES treatment [3, 10, 19]. Despite the prevailing concept of the Warburg effect as a result of an irreversible damage to the oxidative capacity of cancer mitochondria, findings from our group and others [20, 21] indicate that the Krebs cycle is intact in leukemias, and its activity depends on substrates such as Gln and fatty acids to generate intermediates for biosynthetic pathways and to survive oxidative stress through the production of GSH.

Limiting Gln supply caused reduction in the concentration of oncometabolite 2-HG, which is known to play a key role in the pathogenesis of IDH-mutated AML, translating into differential sensitivity to CB-839. Mutant IDH produces 2-HG, which induces histone- and DNA-hypermethylation through inhibition of epigenetic regulators [22–26]. Notably, both accumulation of 2-HG due to IDH-mutant enzymes and the differentiation block was reversed with specific small molecule inhibitors of mutant IDH in both preclinical studies [3, 27, 28] and in ongoing clinical trials [29]. Our preliminary results indicate that similar phenotypes could be observed with CB-839. Notably, CB-839 monotherapy demonstrated signs of clinical efficacy in an ongoing AML trial, causing stable disease in 5 (33%) of 15 efficacy-evaluable elderly AML patients and one complete response with incomplete recovery of peripheral blood cell counts in IDH2-mutated relapsed AML patient [30].

In summary, our findings support the important role of Gln in the metabolic requirements of AML cells, and previously unrecognized role of Gln metabolism in myeloid differentiation of IDH-mutant AML. Although these findings require further validation, overall the data support the feasibility of targeting Gln metabolism with GLS inhibitors in AML, likely requiring combinations with standard and targeted agents.

## MATERIALS AND METHODS

### Chemicals

BPTES was synthesized at Dr Aaron Schimmer's lab and prepared as described previously [6] with a 0.3% final concentration in dimethyl sulfoxide (DMSO). CB-839 was obtained from Calithera Biosciences and was prepared as described by Gross *et al* [3, 33].

### Cell lines and cell culture conditions

The AML cell line panel used in the cell growth assay included NB4 (AML M3), Kasumi (harboring translocation t(8;21)), MV4;11 (MLL), KG-1α KBM5, HL-60, OCI-AML2, OCI-AML3 (mutant *NPM1*), U937, MOLM-13, and MOLM-14 (FLT3-ITD). Acute lymphoid leukemia (ALL) cell lines included REH, NALM-6 (pre-B), Jurkat (T) and Raji (Burkitt). All cell lines were tested and authenticated by The University of Texas MD Anderson Cancer Center Cell Line Validation Core Facility, through the short tandem repeat method.

AML and ALL cell lines were maintained at 37°C under 5% CO_2_ in RPMI 1640 medium (Gibco) supplemented with 10% FBS. Nutrient depletion studies were performed with glucose and Gln-free medium without pyruvate. Reconstituted medium for all experiments was supplemented with 10% FBS; when needed, glucose or Gln was added to the medium at final concentrations of 11 mM and 2 mM, respectively.

Peripheral blood samples from untreated patients with AML were collected during standard diagnostic procedures after informed consent was obtained in accordance with the Institutional Review Board (IRB) regulations of MD Anderson Cancer Center. The study design adhered to the tenets of the Declaration of Helsinki and was approved by the ethics committees of the participating institutions before its initiation. Mononuclear cells were separated from the patient samples by Ficoll-Hypaque (Sigma-Aldrich) density gradient centrifugation. These primary leukemia samples were maintained in StemEZ Serum-Free Medium (Cellagen Technologies).

THP-1 cells were stably transduced with doxycycline-inducible mutant IDH1-R132H or IDH2-R140Q construct as described by Chan *et al*. [16].

### Viability assay and apoptosis detection

AML cell lines and primary cells were incubated in triplicate in their corresponding medium with one of the glutaminase inhibitors or DMSO in a range of concentrations. After 24–120 hours of treatment with BPTES or CB-839 at 37°C in a humidified atmosphere containing 5% CO_2_, these monocultured cells were harvested and resuspended in binding buffer containing annexin V (Roche Diagnostics). Apoptotic cells were detected by annexin V flow cytometry after gating on CD45+ cells. Viable cells were detected and counted by flow cytometry with anti-human CD45-FITC antibody (BD Pharmingen) staining after exclusion of nonviable cells (by diamidino-2-phenylindole [Sigma-Aldrich]) and apoptotic cells. Flow cytometry was performed on a Gallios Flow Cytometer, and data were analyzed by Kaluza Flow Analysis software (Beckman Coulter).

### Western blotting

Cultured or co-cultured cells were counted, collected, and subjected to lysis in lysis buffer (1×10^6^ cells/mL). Total proteins (15 μL) from the lysates were separated by SDS-PAGE and transferred onto Hybond ECL nitrocellulose membranes. Western blotting analyses were performed using the following primary antibodies: 1:1000 diluted GAC/KGA dual-specificity antibody (#ab156876; Abcam) that recognizes an epitope within the *N*-terminal region (between amino acids 72 and 125) and that is common to both endogenous GAC and KGA but is not present in either ΔN-GAC or ΔN-KGA. The analysis also included a commercial diluted anti-GAC (#19958-1-AP; Proteintech Group) that recognizes the unique *C*-terminal tail of GAC, and 1:8000 diluted anti-β-actin (Sigma). Secondary antibodies were chosen according to the species of origin of the primary antibodies and detected by using the Odyssey Imaging System (Li-Cor Biosciences).

### Metabolic analysis

The LC-MS method was described previously by Gross *et al*. [3]. Briefly, leukemia cells and BM-MSC were exposed to BPTES or CB-839 under hypoxic or normoxic conditions. The samples were homogenized in a methanol:water mixture (80:20) containing 10 μmol/L ^13^C_5_, ^15^N-Glu as the internal standard and analyzed for metabolite levels by LC-MS/MS (tandem mass spectrometry) using the SCIEX API4000 (Applied Biosystems).

GC-MS metabolite extraction and analysis was performed at the Metabolomics Core Facility at the University of Utah as described by A Ji-ye *et al*. [37] with minor modifications. Peak intensities of 92 metabolites were obtained for four replicates for each experimental condition. Individual tubes containing cell pellets were exposed to cold (−20°C) 90% methanol. The samples were incubated for 1 hour at −20°C, followed by centrifugation at 30,000*g* for 10 minutes. Each supernatant containing the extracted metabolites was transferred to a new disposable tube and completely dried *in vacuo*. All GC-MS analyses were performed with a Waters GCT Premier mass spectrometer fitted with an Agilent 6890 gas chromatograph and a Gerstel MPS2 autosampler. Dried samples were suspended in 40 μL of a solution of O-methoxylamine hydrochloride in pyridine (40 mg/mL) and incubated for 1 hour at 30°C. The same solution (25 μL) was added to the autosampler vials. N-methyl-N-trimethylsilyltrifluoracetamide (10 μL) was added automatically via the autosampler to each sample, and the samples were then incubated for 1 hour at 37°C with shaking. A fatty acid methyl ester standard solution (3 μL) was added to each sample via the autosampler, and 1 μL of each prepared sample was injected to the gas chromatograph inlet in the split mode, with the inlet temperature held at 250°C. A 5:1 split ratio was used for this analysis. The gas chromatograph had an initial temperature of 95°C for 1 minute followed by a 40°C/min ramp to 110°C and a hold time of 2 minutes. This was followed by a second 5°C/min ramp to 250°C, a third ramp to 350°C, and a final hold time of 3 minutes. A 30 m Phenomex ZB5-5 MSi column with a 5 m guard column was employed for chromatographic separation. Helium was used as the carrier gas at 1 mL/min.

Data were collected by MassLynx 4.1 software (Waters). For first-pass data analysis, the targeted approach for known metabolites was used. Metabolites were identified and their peak area was recorded using QuanLynx. These data were transferred to an Excel spread sheet (Microsoft) and the metabolites identities were established by using a combination of an in house metabolite library developed from pure purchased standards and the commercially available National Institutes of Standards and Technology library. All the data was normalized to D4-Succinate, internal standard.

### Stable isotope-resolved metabolomics (SIRM)

For the SIRM studies [38, 39], ^13^C_5_, ^15^N_2_Gln (Cambridge Isotopes Laboratories, Inc.) or unlabeled Gln was added to the culture medium during 24-hour treatment with CB-839 (1 μM) CB-839 (or DMSO in both normoxic and hypoxic conditions at 37°C in HL-60 cells. Following treatment, 1 mL of the cell-conditioned medium was collected, cells and cell debris were pelleted by centrifugation, and the cell-free medium was snap frozen. Cells were counted and harvested by centrifugation and washed twice with PBS, and cell pellets were snap frozen in liquid nitrogen. All samples were stored at −80°C until extraction.

The polar intracellular metabolites were extracted by dual phase extraction [40, 41] and dried in a CentriVap Concentrator (Labconco) at 4°C. All samples were spiked with the following internal standards at a final concentration of 0.2 ppm: d_4_-alanine, d_3_-serine, d_2_-glycine, d_4_-citric acid, d_5_-Gln, d_3_-malic acid, d_3_-aspartic acid, d_2_-fumaric acid, d_3_-glutamic acid, d_4_-succinic acid, d_5_-tryptophan, d_2_-cysteine, d_4_-cystine, and 1-^13^C-glucose. LC-MS analysis was performed on a Q Exactive Hybrid Quadrupole-Orbitrap mass spectrometer equipped with an Accela 1250 pump and autosampler (Thermo Scientific). An injection volume of 5 μL was used for all samples. Metabolites were separated on a 150×2.1 mm Kinetex 2.6 μm C18 100 Å column (Phenomenex). Mobile phase A consisted of water with 0.2% formic acid. Mobile phase B was HPLC-grade methanol with no additives. Separation was achieved by means of a gradient as follows: 95% mobile phase A for 2 minutes, immediately decreasing to 70% of A, then changing from 70% to 20% over 8 minutes, immediately decreasing to 2% of A and holding for 5 minutes, followed by holding at 95% of A for 15 minutes to re-equilibrate the column. The total run time was 30 minutes with a flow rate of 0.25 mL/min. Eluent entered the ion trap via an electrospray ionization source. The following electrospray ionization source parameters were used for acquisition: spray voltage at 4.0 kV, capillary temperature at 300°C, sheath gas flow at 55 units, and auxiliary gas at 30 units. Solvent blanks and pooled quality controls were injected periodically to monitor column carryover and instrument stability. Data were analyzed by Xcalibur Software (Thermo Scientific). Peak intensities were normalized to the corresponding deuterated standard and to cell number to calculate the relative metabolite levels under different treatment conditions.

### Seahorse oxygen consumption rate and extracellular acidification rate

Oxygen Consumption Rate (OCR) and extracellular acidification rate were determined using a Seahorse Bioscience XF96 Extracellular Flux Analyzer according to the manufacturer's protocol. OCI-AML3 cells were treated with DMSO, BPTES inhibitor (20μM) or CB-839 (1μM) for 12 hours in RPMI medium containing 10% FBS. Cells were washed with PBS, resuspended in XF Assay medium, immediately plated onto XF96e Seahorse Biosciences plates pre-coated with Cell-Tak BD at a density of 0.2–0.3×10^6^ cells per well and spun down on the plate to allow cells to adhere equally.

### Differentiation assay

The cell differentiation assay was modified from that described by Wang *et al*. [28]. In brief, mononuclear cells derived from AML patients' peripheral blood samples were incubated at 1–2×10^6^ cells/mL in Iscove modified Dulbecco medium supplemented with cytokines (receptor-type tyrosine-protein kinase FLT3, 50 ng/mL; stem cell factor, 50 ng/mL; thrombopoietin, 10 ng/ml; IL-3, 3 ng/mL; IL-6, 5 ng/mL; and erythropoietin, 2 unit/mL) and treated with DMSO, IDH1 inhibitor AGI-5198 (0.5 μM), IDH2 inhibitor AGI-6780 (0.5 μM), or CB-839 (0.111 μM or 1 μM) for 8 days. Cells were spun down, and fresh medium was added at day 4 of differentiation. The differentiation was assessed by flow cytometry analysis measuring changes in cell surface markers associated with granulocytic differentiation (CD11b-APC) and monocytic differentiation (CD14-PB; both from Bio Legend. Additionally, morphologic features of differentiation were assessed after specific culture conditions on samples prepared by cytospin for hematoxylin and eosin staining.

### The cancer genome atlas AML RNAseq data analysis

TCGA AML RNASeqV2 level 3 data and clinical information were downloaded on April 3, 2014. The raw expression signal of a gene was used to form the gene expression data matrix. The RNASeq read count data were normalized using the TMM method [42] implemented in the Bioconductor edgeR software package [43]. Gene expression values shown are the means (log2-RPKM). We analyzed gene expression of *GLS* (glutaminase [alias *GLS1*], Gene ID: 2744), *GLS2* (glutaminase 2, Gene ID: 27165), and *GLUD1* (glutamate dehydrogenase 1, Gene ID: 2746). To obtain comparable gene expression and isoform expression values, we used TCGA normalized data for both gene and isoform, followed by log2-transformation by the MD Anderson Genome Data Analysis Center batch-effect group. *GLS1*, *GLS2*, and *GLUD1* expression levels were continuously unimodal and were treated as continuous variables [42, 43].

### Microarray-based gene expression profiling and analysis

The expression of *GLS* and *GLUD1* genes was determined oligonucleotide microarrays (HG-U133 Plus 2.0, Affymetrix) in 288 AML samples comprising all cytogenetic groups and in 103 normal karyotype BM samples (leukemia) from the MLL as described by Haferlach *et al* [13]. All AML samples were obtained from untreated patients at the time of diagnosis. Cells used for microarray analysis were collected from the purified fraction of mononuclear cells after Ficoll density centrifugation. The analysis was conducted at log2-transformed gene expression intensities. For each gene, ANOVA was used to compare gene expression values between different cytogenetic categories. The Wilcoxon test was used for pairwise comparison between each pair of cytogenetic categories.

Expression of genes related to Gln metabolism was evaluated in a separate patient cohort described by (Verhaak *et al*. *n* = 536, Affymetrix HG-U133A) [14]. Associations between gene expression levels and AML subtypes were tested in a multiple linear regression model. Individual regression coefficients belonging to the distinct AML subtypes were tested with a *t*-test for each probe set. A p-value ≤0.05 was considered significant.

## SUPPLEMENTARY FIGURES AND TABLES


