# Supplementary material for: Inhibiting glutaminase in acute myeloid leukemia: metabolic dependency of selected AML subtypes
Source: Oncotarget. 2016 Oct 27;7(48):79722–35. doi: 10.18632/oncotarget.12944 (PMC5340236; doi:10.18632/oncotarget.12944)
Supplement: Supplementary file 1 [file oncotarget-07-79722-s001.pdf]

Inhibiting glutaminase in acute myeloid leukemia: metabolic dependency of selected AML subtypes

SUPPLEMENTARY FIGURES AND TABLES

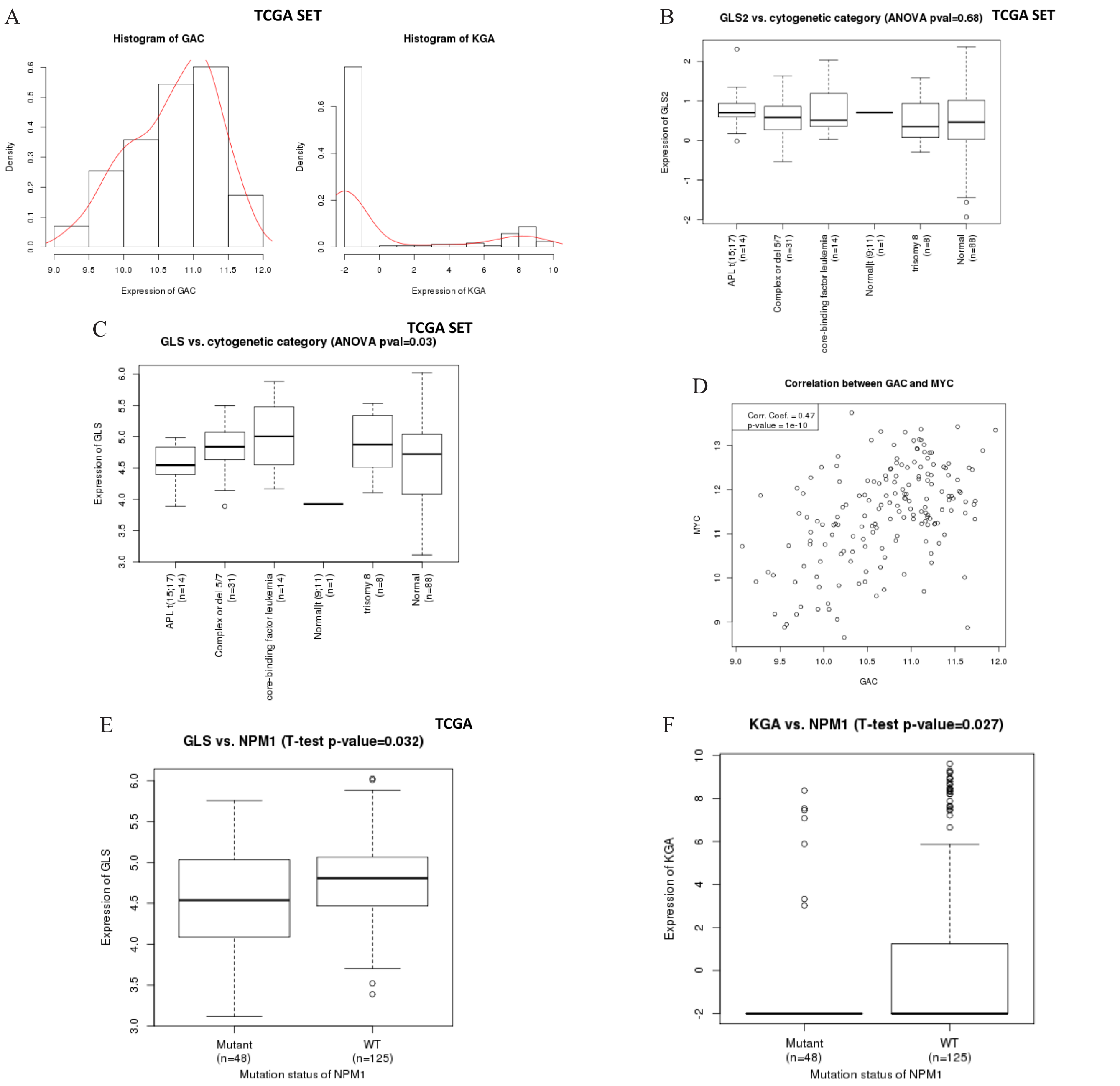

Supplementary Figure S1: *GLS* isoforms expression varies in different cytogenetic categories of AML. A. Expression values (log2-normalized counts) of two isoforms of *GLS* (*GAC* and *KGA*). **B.** Gene expression values of *GLS2* (batch-effect adjusted, normalized, log2-transformed RPKM (Reads per Kilobase of transcript per Million mapped reads) values) in different AML cytogenetic abnormality categories. Only samples whose cytogenetic category was known were used in this comparison (i.e., excluding category "Not Available"). The p-value was determined by ANOVA. **C.** Boxplot comparing expression values of *GLS* from theTCGA AML dataset. **D.** Correlation of expression values between genes *GAC* and *c-MYC*. The Pearson correlation coefficient and p-value of the correlation test are shown in the plot. **E.** Expression of *GLS* mRNA by mutation status of gene *NPM1* **F.** and of *KGA* by mutation status of gene *NPM1*. A *t*-test was used to test the difference in mRNA expression values between mutation groups.

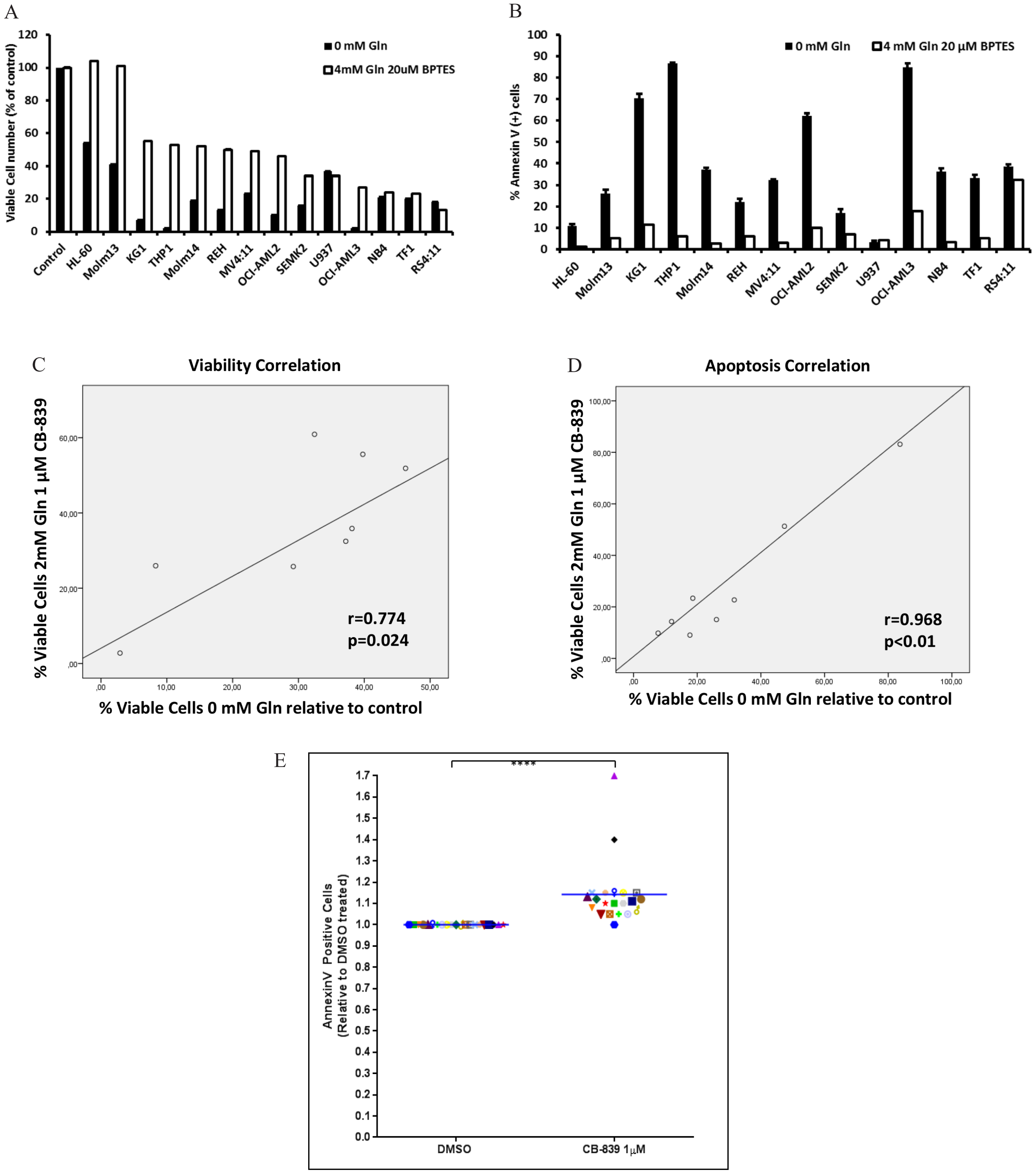

Supplementary Figure S2: Gln deprivation and GLS1 inhibition by BPTES decrease viable cell number and induce apoptosis in leukemic cell lines. A. The percentage of viable CD45+ cells normalized to viable CD45+ cells in control samples was measured after 3 days by using multicolor flow cytometry. **B.** Levels of specific apoptosis in cells deprived of Gln or treated with glutaminase inhibitor BPTES relative to controls, measured by annexing V flow cytometry. (C-D) Correlation between percentages of **C.** viable cell counts relative to controls and **D.** apoptosis after CB-839 treatment plotted on y-axis; glutamine withdrawal is plotted on the x-axis. Each data point depicts the mean value for an individual cell line. The correlation coefficient and p-value are shown in the plot. The line represents the regression fit. **E.** Average annexin V values in primary AML cells after treatment with DMSO or CB-839 (relative to DMSO).

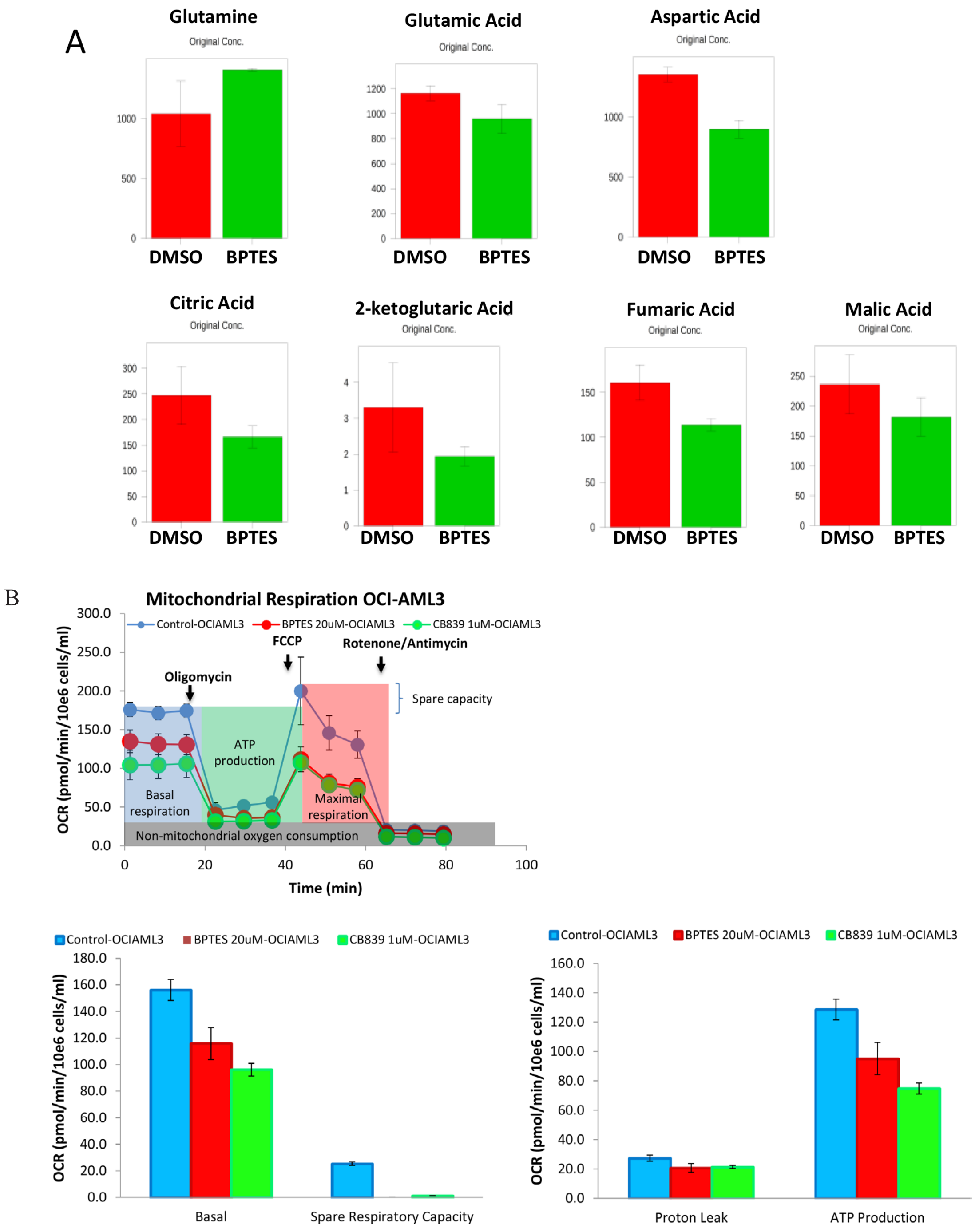

Supplementary Figure S3: BPTES inhibits Gln utilization, TCA cycle activity and reduces oxygen consumption in AML cells. A. GC-MS–based metabolomics analysisof OCI-AML3 cells treated with BPTES (20 µM) for 24 hours compared to DMSO-treated controls. Semi-quantitative levels of the TCA cycle intermediates citrate, fumarate and succinate were significantly decreased by glutaminase inhibition in leukemic cells. BPTES decreased Gln and asparagine catabolism, shown by the decrease in the relative concentrations of intracellular Glutamate, and aspartate. Peak intensities of 92 metabolites were obtained for four replicates of cultured OCI-AML3 treated with DMSO or 20 µM BPTES. The values were averaged, normalized by the median, and log-transformed and scaled by subtracting the mean and dividing by standard deviation; bars, standard deviation. P-values were calculated by one-way ANOVA. Statistical analysis was performed using Metaboanalyst software [43]. **B.** To characterize the contribution of Gln to the TCA cycle, effects of GLS inhibitor CB-839 and BPTES on OCR was determined using a Seahorse Bioscience XF96 Extracellular Flux Analyzer. Pretreatment with CB-839 (1 µM) or BPTES (20 µM) for 12 hours caused a decrease in basal OCR, reduced ATP production, and decreased maximal respiratory capacity (after the mitochondrial uncoupler FCCP was added) in OCI-AML3 cells.Six replicate wells (3 $\times 10^5$ cells per well) were analyzed. Representative example of one out of four independent experiments is shown.

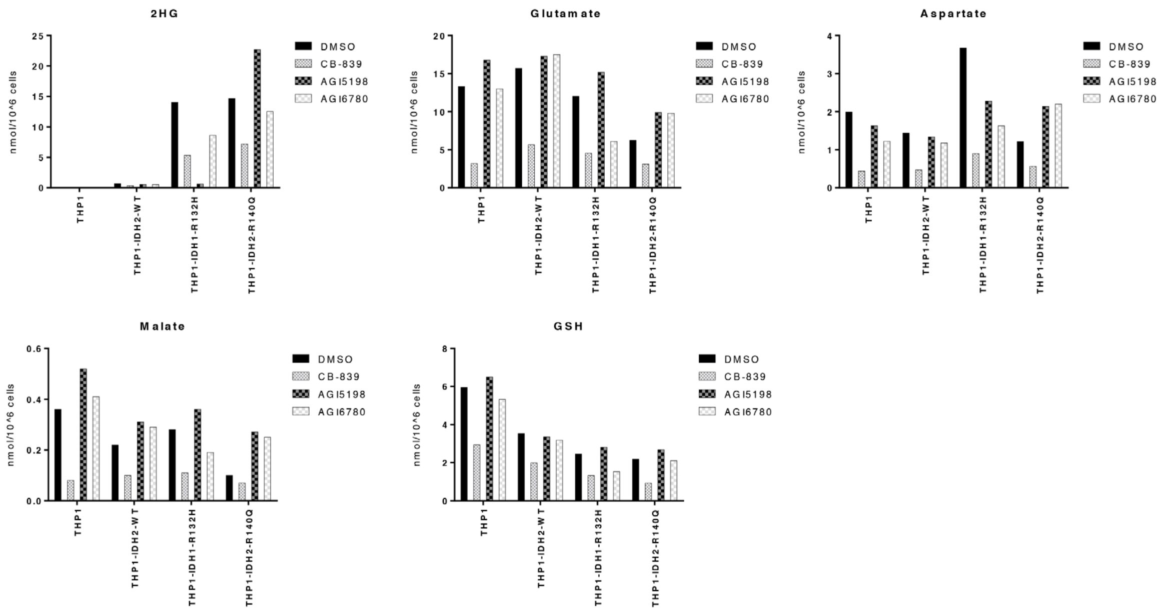

Supplementary Figure S4: CB-839 treatment decreases cellular levels of 2-HG, glutamate, aspartate, TCA acid cycle intermediates, and GSH in *IDH1/IDH2*-mutant THP1 cells. Intracellular metabolite levels were measured in *IDH1/IDH2*-mutant THP1 cells treated with CB-839 (1 µM), AGI-5198 (500 nM), or AGI- 6780 (500 nM) for 96 hours.

Supplementary Table S1: Mean expression values and corresponding percentiles of most frequently expressed genes in the AML dataset from TCGA

Gene Symbol	Entrez ID	Mean Expression value	Percentile
GLS	2744	12	96
GLUL	2752	14	100
GLUD1	2746	11	94
GOT2	2806	10	83
GPT2	84706	9	66

GLS GENE: Glutaminase.
GLUL GENE: Glutamate-ammonia ligase.
GLUD1 GENE: Glutamate dehydrogenase.
GOT2 GENE: Glutamic-oxaloacetic transaminase 2.
GPT2 GENE: Glutamic-pyruvic transaminase 2.

Supplementary Table S2: Cell line characteristics

CELL LINE	CYTOGENETICS
NB4	t(15;17)
Kasumi	t(8;21)
MV4-11	t(4;11)
KG-1	t(4;8), t (8;12)
KBM5	t(9;22)
HL-60	Complex Karyotype t(5;7), t(5;16)
OCI-AML2	Complex Karyotype
Raji	t(8;14)
OCI-AML3	t(1;18)
Jurkat	t(5;10)

Supplementary Table S3: Patient sample information

PATIENT SAMPLES (UPIN)	SOURCE	BLASTS (%)	MOLECULAR	CYTOGENETICS
PS1 4064236	PB	88	GATA2	complex incl del17(p11.2)
PS2 6053718	PB	99	AML-ETO fusion, N-RAS and TET2 mutation	t(8;21)+complex
PS3 4015354	PB	39	CBFB-MYH11 fusion	Inv 16
